# Feasibility study on cardiac resynchronization in the treatment of heart failure by single left bundle branch pacing

**DOI:** 10.1038/s41598-023-48820-z

**Published:** 2023-12-14

**Authors:** Yadong Du, Lijin Pu, Baotong Hua, Yanzhou Lu, Xiuli Wang, Ling Zhao

**Affiliations:** 1 The First Affiliated Hospital of Kunming Medical University, Kunming, 650100 China; 2 University of South Dakota, Vermillion, SD 57069 USA; 3 Yunnan College of Business Management, Kunming, 6500032 China

**Keywords:** Cardiology, Cardiac device therapy

## Abstract

To examine the feasibility of single left bundle branch pacing for cardiac resynchronization therapy (CRT) by carrying out a frequency adaptive atrioventricular delay (RAAVD) algorithm and automatic optimization of the single left bundle branch pacing atrioventricular interval (AVI) based on the right atrioventricular interval (RAS–RVS). Thirty-six patients with CRT class Ia indications according to the European Society of Cardiology 2016 guidelines and implanted with RAAVD functional three-chamber pacemakers were prospectively enrolled in this study. Patients were divided into a single left bundle branch pacing group (n = 21) and a standard biventricular pacing group (n = 15). The optimization of the two groups was performed under standard cardiac colour Doppler ultrasound, followed by the comparison of the QRS width, cardiac function improvement, and echocardiography indicators. The ratio of AVI to the right atrial-right ventricular interval (RAS–RVS) after single LV pacing optimization was defined as the single left bundle branch pacing coefficient (LUBBPε). In comparison to the BVP, the QRS was significantly narrowed (*P* = 0.017), accompanied by a significantly increased proportion of patients with NYHA class I and II, as well as the 6MWT. Compared with standard biventricular pacing, LVEDD was significantly shortened (*P* = 0.045), accompanied by significantly improved LAD, AVVTI, EA distance/RR, IVMD, and TS-SD after the operation. RAS–RVS was 156 ± 33 ms, the optimized AVI was 102 ± 10 ms, and LUBBPε was calculated to be 0.66 ± 0.06. Depending on the LUBBPε, a three-chamber pacemaker with a single left bundle branch pacing system was developed based on RAS–RVS-optimized AVI automatically. A three-chamber pacemaker with single left bundle branch pacing can achieve CRT based on RAS–RVS, reaching the optimal AVI of 66% of RAS–RVS.

## Introduction

Cardiac resynchronization therapy (CRT) serves as a critical treatment strategy for patients with symptomatic heart failure, left ventricular (LV) dysfunction and cardiac electrical desynchrony^1,2^. However, approximately 30–40% of patients still fail to respond well or even do not respond to conventional biventricular pacing during treatment. In comparison to His bundle pacing (HBP), left bundle branch pacing (LBBP), as an alternative pacing therapy to His-Purkinje system pacing, can eliminate multiple downsides of HBP and provides a low and stable threshold with a unique correction of the distal conduction system benefitting from the anatomical and electrical characteristics^3–6^. In fact, there is sufficient evidence that fusion pacing, in which cardiac conduction is transmitted down the atrioventricular node (AVN) to the right ventricle to fuse with LV pacing, can achieve the best haemodynamic stability and finally achieve cardiac synchronization.

In fact, there is sufficient evidence that fusion pacing, in which sinus rhythm is transmitted down the atrioventricular node (AVN) to the right ventricle to fuse with LV pacing, can achieve the best haemodynamic stability and finally achieve cardiac synchronization^7–14^. Left univentricular pacing (LUVP) even serves better than, or at least not worse than, BVP^15–18^. Physiologically, the atrial-ventricular delay (AVD) will vary dynamically with the change in heart rate to achieve the filling effect of the atrium on the ventricle and elevate ventricular ejection, posing a serious challenge to fusion pacing. LUVP can be completed through AVD, which can provide optimal fusion between sinus rhythm down the atrioventricular node (AVN) to the right ventricle and LV pacing^8^.

Adaptive CRT algorithm: This algorithm can achieve the dynamic optimization of CRT pacing (BiV vs. LV) and AV/VV intervals. If the intrinsic AVD is normal and without a heart rate exceeding 100 beats per minute, the algorithm provides optimized single LV pacing with an AVI equal to 70% of the intrinsic AVD, in which LV pacing can be fused with RV excitatory revealing more stable haemodynamics^19,20^. Depending on the above algorithm, LUVP can realize CRT. However, the adaptive CRT algorithm works based on traditional CRT, in which LV electrodes are generally implanted in the lateral or posterior LV veins, accompanied by an AVI equal to 70% of the intrinsic AVD. When the implantation of the LBBP LV electrode is below the LBBB point and located in the conduction system region of the heart itself, the transformation in the ratio of the optimal AVI to the inherent AVD will also occur, with the specific value unproposed. The aim of this study was to investigate whether single left ventricular pacing in the left bundle branch area is noninferior to BVP in terms of QRS duration, cardiac function, and echocardiographic parameters. A new concept is proposed. The ratio of AVI optimized by single left bundle branch pacing to the right atrioventricular interval (RAS–RVS) was defined as the single left bundle branch pacing coefficient (LUBBPε), which was the ratio of the optimal sensed atrioventricular interval (SAV) to the intrinsic atrioventricular interval (the atrioventricular interval measured when sinus rhythm is transmitted through the atrioventricular junction to the ventricle without pacing). The aim of this study was to develop an automatically optimized single left bundle branch pacing system for a three-chamber pacemaker. This system can automatically optimize the physiological state of the atrioventricular interval (RA–RV) according to the single left bundle branch pacing coefficient (LUBBPε).

## Methods

### Study population

Thirty-six patients with chronic heart failure and left ventricular ejection fraction (LVEF) ≤ 35%, sinus rhythm, complete left bundle branch block (LBBB), QRS duration ≥ 150 ms, NYHA class II–IV under optimal medical therapy were enrolled in this study. Thirty-six patients signed informed consent to be implanted with a three-chamber pacemaker with rate-adaptive atrioventricular delay (RAAVD) function. The patients were matched by age, sex, QRS duration, NYHA functional class and 6-min walk test. According to the individual differences and specific intraoperative conditions, the patients were divided into a single left bundle branch pacing group (21 patients) and a standard biventricular pacing group (15 patients). Each patient was optimized before the operation and 1, 3, 6, and 12 months after the operation, and the last follow-up time prevailed. Patients were excluded if they had any of the following conditions: (1) atrioventricular block; (2) right bundle branch block; (3) patients with a life expectancy less than 1 year; (4) patients with potentially reversible cardiomyopathy; (5) patients undergoing heart transplantation or waiting for heart transplantation; (6) patients with cardiac valves; (7) patients with hypertrophic obstructive cardiomyopathy; and (8) patients unable to participate in the follow-up. The study was approved by the institutional ethical review board of the hospital. All methods in this study were performed in accordance with relevant guidelines and regulations.

#### Equipment and materials

All three chamber pacemakers were from Medtronic. The models are as follows: Synacra C2TR01 CRT, Maximo II CRT-D, Insync Sentry 7298 CRT-D, Egida D394TRG CRTD, Brave DTBC2QQ, and D7BC2D1 CRTD. DTBA2D4/D1 CRTD.

### Indicators

#### Measurement methods of QRS complexes

In this study, the QRS complex was measured from the end of the pacing pulse to the end of the QRS complex, namely, S-QRS.

#### Ultrasonic cardiogram

The ultrasound and synchronization indexes in both groups were measured before the operation and 1 month, 3 months, 6 months and 12 months after the operation. Normal cardiac ultrasound indicators referred to left ventricular ejection fraction (LVEF), left atrial dimension (LAD), left ventricular end diastolic dimension (LVEDD), and aortic valve velocity time integral (AVVTI). The synchronization index included the following: (1) Atrioventricular synchronicity: EA distance/RR; (2) Interventricular synchrony: Interventricular mechanical delay time (IVMD); (3) Left intraventricular contractive synchrony: standard deviation of time intervals of the 12 LV segments (TSI-12S-D).

#### Preoperative Holter examination

**Figure 1:**
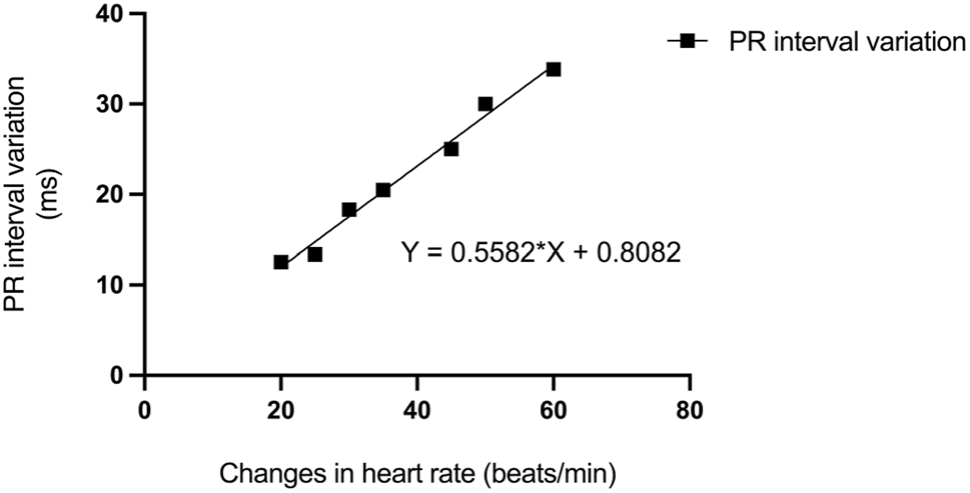
Regression equation of the relationship between the change of heart rate and the change of PR interval in one patient in the single left bundle branch pacing group.

Preoperative 24-h Holter examination was performed. PR interval was measured at HR 60, 65, 70... and the highest heart during sinus rhythm. Taking heart rate as the independent variable (x) and the change of PR interval as the dependent variable (y), the function y = ax + b was established to calculate the change of PR interval from the change of heart rate as the basis for setting the frequency adaptive AV to ensure the real-time and accurate tracking of the physiological PR interval of the dynamic changes of the right. In this way, the LV pacing can form an optimal fusion wave with the excitation transmitted down from the right His-Purkinje system to realize the CRT. Note: a is a constant term and b is a standardized partial regression coefficient. The PR interval corresponding to the lower rate interval (LRI) and upper rate interval (URI) can be derived according to this formula, which can serve as the basis for setting the parameters of RAAVD (Fig. 1).

#### Assessment of cardiac function

NYHA and 6MWT were used to evaluate preoperative and postoperative cardiac function.

### Programming and optimization

#### Parameter optimization of the BVP group

After the operation, the BVP was set. AVI was titrated bidirectionally (prolonged or shortened) with a step size of 10 ms, of which the most optimized was obtained with the smallest MRA and IVMD, the largest AVVTI and the narrowest QRS wave.

#### Parameter optimization of the single left bundle branch pacing group

The three-chamber pacemaker was programmed for single LV pacing, in which only sensing and defibrillation functions were retained, and the VS was set to the right ventricle by default. The default atrial sense compensation (ASC) was 30 ms. The atrioventricular interval (AVI) was prolonged until the intracardiac maps are showing AS-VS indicated, and the AS-VS interval (RA–RV) interval was measured. Based on the ASC subtracted from the interval of AS-VS (RA–RV), bidirectional titration was performed in 10 ms steps, and the titrated AVI was shown under echocardiography. When the LVEF and AVVTI were the largest and the MRA was the smallest, and QRS was narrowest, it was the optimal AVI, that is, the best AVI of the left atrioventricle at a specific heart rate (generally, during optimization, the patient’s heart rate is greater than the lower limit of pacing, which is the sensing AV, or SAV). The single left bundle branch pacing coefficient (LUBBPε) was calculated. The average of the two results was used as the single left bundle branch pacing coefficient in this patient and was recorded.

#### Parameter settings

The start rate was set as the lower limit rate (LRI) of the pacemaker, and the stop rate was set as the upper limit rate URI of the pacemaker. The RAAVD parameters were set according to the PR interval at different heart rates of Holter collected before operation or the regression equation of the relationship between heart rate change and PR interval established by Holter. SAV interval for start rate/at stop rate = optimal AVI+ (PR interval for start rate/at stop rate − optimized PR interval). In terms of three-chamber pacemakers, the optimized RAS–RVS can be adopted instead of the optimized PR interval. PAV = SAV + ASC. When RAAVD is turned on, the atrioventricular interval of the pacemaker can be dynamically set according to the RAAVD parameters to synchronize left and right ventricular electrical activation, complete fusion pacing, and realize CRT.

### Statistical analysis

Data were analysed using SPSS 22.0 for Windows (SPSS Inc., Chicago, IL). When the numerical data conformed to normality, the mean (standard deviation) was used to describe the homogeneity of variances and was compared with the T test. Otherwise, the data are described by the median with the maximum and minimum, and the nonparametric test was used. The chi-squared test was used for enumeration data. For all statistical analyses, *P* < 0.05 after correction was considered a significant difference.

### Ethics approval

The study protocol was reviewed and approved by the Medical Ethics Committee of the First Affiliated Hospital of Kunming Medical University in accordance with the 1964 Declaration of Helsinki and its later amendments.

## Results

### Follow-up

No cardiovascular death occurred during the follow-up period (Table 1).

**Table 1:** Baseline characteristics of the patients.

Measurements	SLBBP (n = 21)	BVP (n = 15)	*P* values
Male/female	15/6	11/4	1.000
Age (years)	65.95 ± 100.20	57.84 ± 140.20	0.055
Follow-up time(range, months)	6 (6–12)	6 (3–12)	0.071
NYHA II (%)	6 (28.6)	5 (33.3)	0.931
NYHA III (%)	11 (52.4)	6 (40.0)	
NYHA IV (%)	4 (19.0)	4 (26.7)	
6MWT/m	374.23 ± 80.10	365.07 ± 84.36	0.742
QRS duration/ms	169.05 ± 19.69	162.87 ± 30.30	0.895
LVEF/%	30.0 ± 6.39	25.80 ± 6.44	0.061
LAD/mm	41.67 ± 6.03	43.87 ± 9.73	0.070
LVEDD/mm	67.81 ± 9.50	70.53 ± 8.80	0.875
AVVTI/ms	17.47 ± 6.56	17.47 ± 3.79	0.999
EA distance/RR	0.26 ± 0.06	0.27 ± 0.09	0.665
IVMD/ms	64.57 ± 18.70	64.40 ± 32.00	0.985
TSSD-12/ms	138.95 ± 19.31	145.40 ± 38.31	0.556

SLBBP, Sing left bundle branch pacing; BVP, Biventricular pacing; NYHA, New York Heart Association; 6MWT, 6-min walk test; LVEF, left ventricular ejection fraction; m, meter; mm, millimetre; ms, millisecond.

### Comparison of each index between the single left bundle branch pacing group and BVP group

#### Electrocardiogram and clinical parameters

**Figure 2:**
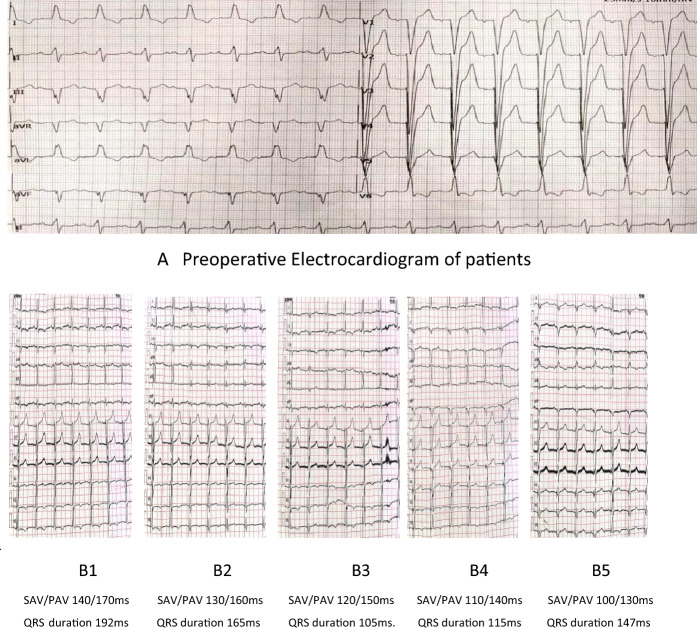
Preoperative electrocardiogram and postoperative optimization were performed in 1 patient with single left bundle branch pacing. (**A**) The patient’s preoperative electrocardiogram showed complete left bundle. branch block with a QRS duration 155 ms. (**B**) Postoperative optimized ECG of the patient. During optimization, the 12-lead. ECG was traced to extend the AVI to the intracavitary map showing as-vs, and titrated bidirectively at 10 ms steps to the narrowest QRS duration of 105 ms. The change of RAAV was set to enter the clinical follow-up.

**Figure 3:**
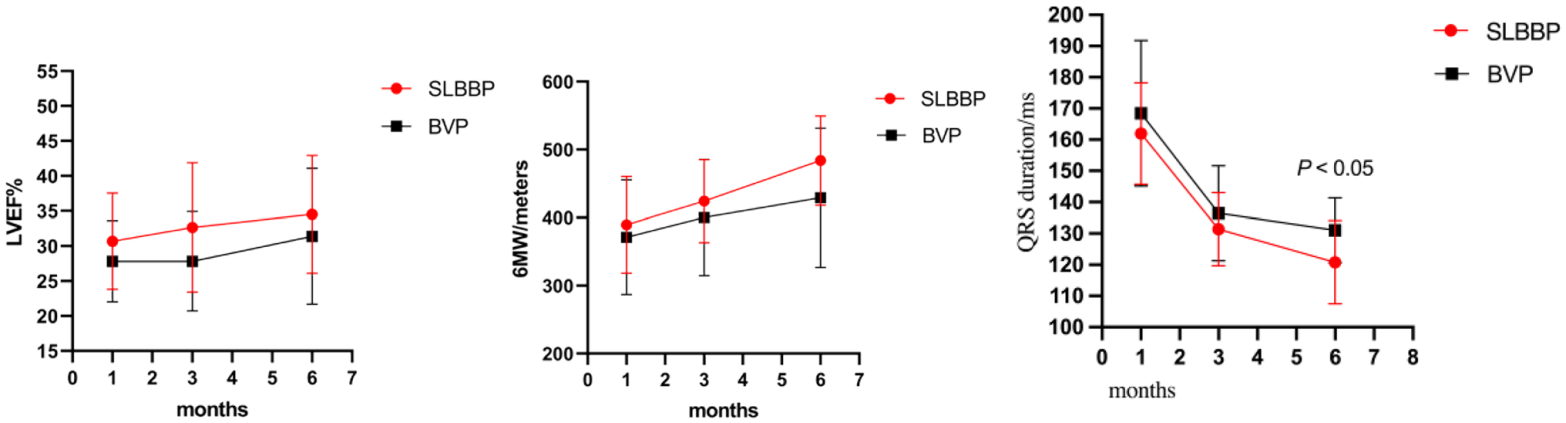
Comparison of postoperative 6-MWT, LVEF and QRS duration between single left bundle branch pacing group and standard biventricular pacing group.

In comparison to the BVP group, the QRS complex was significantly narrowed, accompanied by a significantly increased proportion of patients with NYHA class I and II, as well as the 6MWT (Tables 2, 3, Figs. 2, 3).

**Table 2:** Comparison of preoperative and postoperative clinical indicators and QRS wave between single left bundle branch pacing group and standard biventricular pacing group.

Group	Measurements	Preoperative	Postoperative	*P* values
SLBBP (n = 21)	QRS duration/ms	164.05 ± 19.69	120.81 ± 13.33	0.000
	6MWT/m	374.24 ± 80.10	483.76 ± 65.63	0.000
	NYHAI (%)	0 (0)	4 (19)	0.001
	NYHAII (%)	6 (28.6)	11 (52.4)	
	NYHAIII (%)	11 (52.4)	6 (28.6)	
	NYHAIV (%)	4 (19.0)	0 (0)	
BVP (n = 15)	QRS duration/ms	164.87 ± 19.69	131.00 ± 10.41	0.001
	6MWT/m	369.07 ± 84.36	429.13 ± 102.44	0.072
	NYHAI (%)	0 (0)	2 (13.33)	0.052
	NYHAII (%)	5 (33.33)	5 (33.33)	
	NYHAIII (%)	6 (40.00)	8 (53.34)	
	NYHAIV (%)	4 (26.67)	0 (0)	

SLBBP, Sing left bundle branch pacing; BVP, Biventricular pacing; NYHA, New York Heart Association; 6MWT, 6-min walk test; m, meter; ms, millisecond.

**Table 3:** Comparison of postoperative clinical indicators and QRS duration between the single left bundle branch pacing group and the standard biventricular pacing group.

Measurements	SLBBP (n = 21)	BVP (n = 15)	*P* values
QRS duration/ms	120.81 ± 13.33	131 ± 10.41	0.017
6MWT/m	483.76 ± 65.63	429.13 ± 102.44	0.063
NYHAI (%)	4 (19)	2 (13.33)	0.186
NYHAII (%)	11 (52.4)	5 (33.33)	
NYHAIII (%)	6 (28.6)	8 (53.34)	

SLBBP, Sing left bundle branch pacing; BVP, Biventricular pacing; NYHA, New York Heart Association; 6MWT, 6-min walk test; m, meter; ms, millisecond.

#### Echocardiography

**Figure 4:**
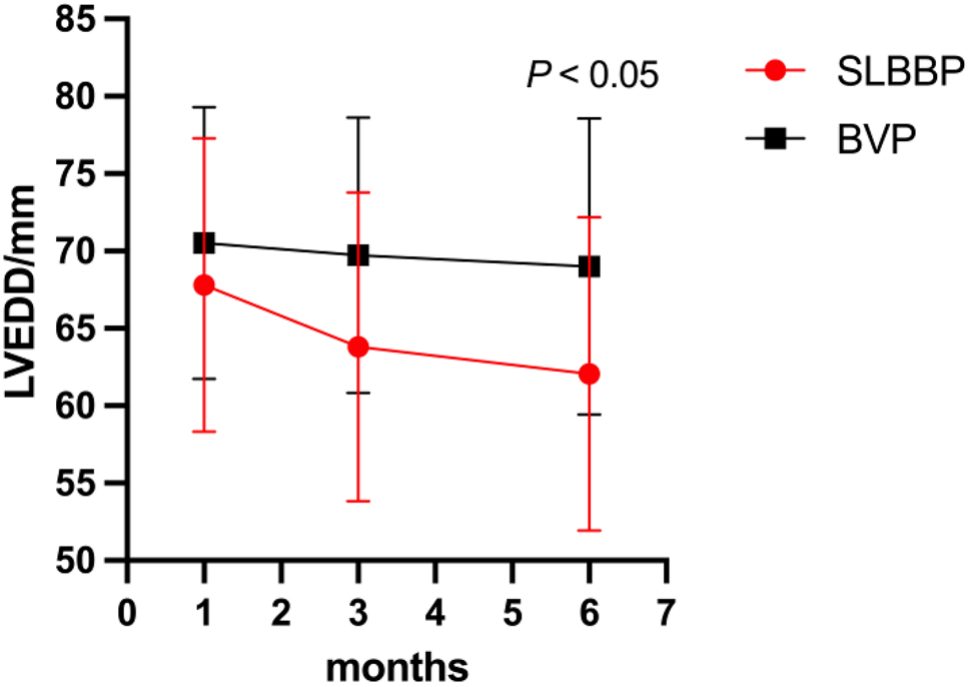
Comparison of postoperative LVEDD between single left bundle branch pacing group and standard biventricular pacing group.

**Figure 5:**
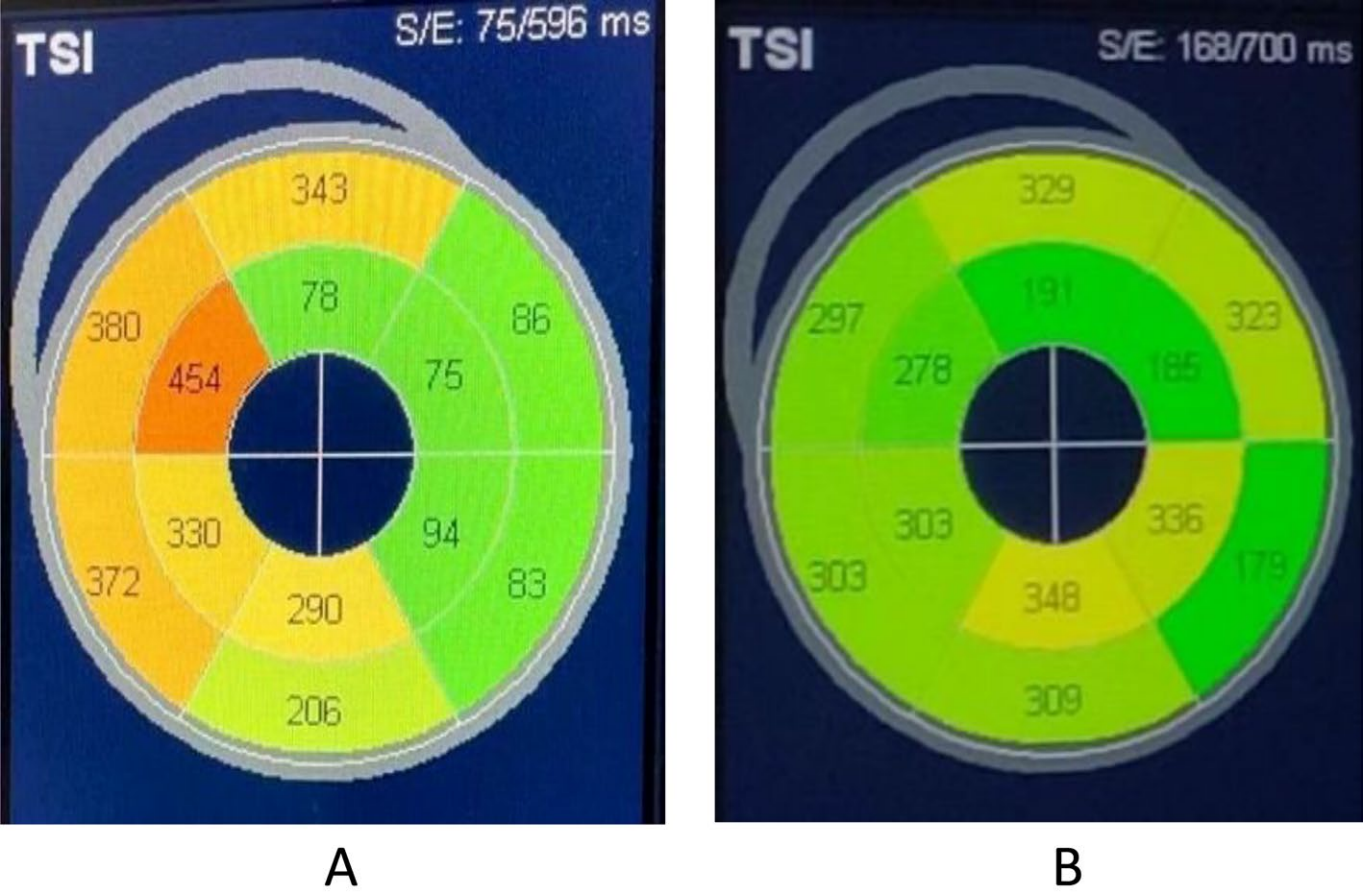
The standard deviation of left ventricular peak time in 12 stages was analyzed by TSI before and after operation. The left ventricular synchrony was significantly improved after surgery, and the. LVEF increased from 34 to 54%. In A, the TSSD-12 was 144 ms preoperatively and in B, the TSSD-12 was 61 ms 10 months postoperatively.

Compared with BVP, LVEDD was significantly shortened, accompanied by significantly improved LAD, AVVTI, EA distance/RR, IVMD, and TS-SD after the operation in the single left bundle branch pacing group (Tables 4, 5, Figs. 4, 5).

**Table 4:** Comparison of preoperative and postoperative echocardiography between single left bundle branch pacing group and standard biventricular pacing group.

Group	Measurements	Preoperative	Postoperative	*P* values
SLBBP (n = 21)	LVEF/%	30.00 ± 6.39	36.42 ± 12.68	0.044
	LAD/mm	41.67 ± 6.03	37.95 ± 6.66	0.065
	LVEDD/mm	67.81 ± 9.50	62.05 ± 10.12	0.064
	AVVTI/ms	17.47 ± 6.56	23.17 ± 5.94	0.005
	EA distance /RR	0.26 ± 0.06	0.34 ± 0.07	0.001
	IVMD/ms	64.57 ± 18.70	37.95 ± 18.69	0.000
	TSSD-12/ms	138.95 ± 19.31	104.33 ± 20.50	0.000
BVP (n = 15)	LVEF/%	25.80 ± 6.44	31.40 ± 9.70	0.073
	LAD/mm	43.87 ± 9.73	38.87 ± 6.70	0.112
	LVEDD/mm	70.53 ± 8.80	69.00 ± 9.55	0.651
	AVVTI/ms	17.47 ± 3.79	23.60 ± 4.10	0.000
	EA distance /RR	0.27 ± 0.09	0.30 ± 0.06	0.352
	IVMD/ms	64.40 ± 32.00	43.27 ± 24.82	0.053
	TSSD-12/ms	145.40 ± 38.31	122.87 ± 35.64	0.106

SLBBP, Sing left bundle branch pacing; BVP, Biventricular pacing; LAD, Left atrial dimension; LVEDD, Left ventricular end diastolic dimension; AVTTI, Aortic valve velocity time integral; EA distance, Mitral flow spectrum from onset of peak E to end of peak A; IVMD, Interventricular mechanical delay time; TSSD-12, The standard deviation of Ts of 12 lefts; mm, millimeter; ms, millisecond.

**Table 5:** Comparison of postoperative echocardiography between the single left bundle branch pacing group and the standard biventricular pacing group.

Measurements	SLBBP (n = 21)	BVP (n = 15)	*P* values
LVEF/%	36.42 ± 12.68	31.40 ± 9.70	0.206
LAD/mm	37.95 ± 6.66	38.87 ± 6.70	0.688
LVEDD/mm	62.05 ± 10.12	69.00 ± 9.55	0.045
AVVTI/ms	23.17 ± 5.94	23.60 ± 4.10	0.809
EA distance /RR	0.34 ± 0.07	0.30 ± 0.06	0.103
IVMD/ms	37.95 ± 18.69	43.27 ± 24.82	0.468
TSSD-12/ms	104.33 ± 20.50	122.87 ± 35.64	0.085

SLBBP = Sing left bundle branch pacing; BVP = Biventricular pacing; LAD = Left atrial dimension; LVEDD = Left ventricular end diastolic dimension; AVTTI = Aortic valve velocity time integral; EA distance = Mitral flow spectrum from onset of peak E to end of peak A; IVMD = Interventricular mechanical delay time; TSSD-12 = The standard deviation of Ts of 12 lefts.

### Single left bundle branch pacing coefficient and automatic optimization algorithm

#### Single left bundle branch pacing coefficient

Automatic optimization algorithm of single left bundle branch pacing based on RA–RV interval in three-chamber pacemaker. LUBBPε = optimized AVI (SAV)/ RAS–RVS  = 0.66 ± 0.06.

#### Automatic optimization algorithm of single left bundle branch pacing based on the RA–RV interval in a three-chamber pacemaker

The results demonstrated that CRT can be achieved by optimizing AVI depending on the RAS–RVS interval in a three-chamber pacemaker with single left bundle branch pacing, with the optimal AVI being 66% of the RAS–RVS interval. Based on the current V–V interval program of three-chamber pacemakers, one step per 10 ms, a three-chamber single-left bundle branch pacing system with automatic AVI optimization was developed, with 0.03 suitable for ε. Accordingly, it was proposed to develop a single-left bundle branch pacing system for a three-chamber pacemaker to automatically optimize AVI based on the RA–RV interval.

The pacing system was composed of (1) an algorithm to derive the right atrial-right ventricular (RA–RV) interval based on the atrial-interatrial phase; (2) optimized LUBBPε (default value: 0.66); and (3) the algorithm to optimize the pacemaker AVI based on the RA–RV interval with AVI (n) = RA(n) − RV(n) interval × LUBBPε. The regression equation of the RA–RV interval was derived based on the AS–AS interval: RA(n) − RV(n) interval = a + b[AS(n − 1) − AS(n)] interval, and the optimal AVI based on the RA–RV interval can be automatically calculated according to the optimized ε (default value 0.66), namely, AVI(n) = RA(n) − RV(n) interval × LUBBPε.

**Figure 6:**
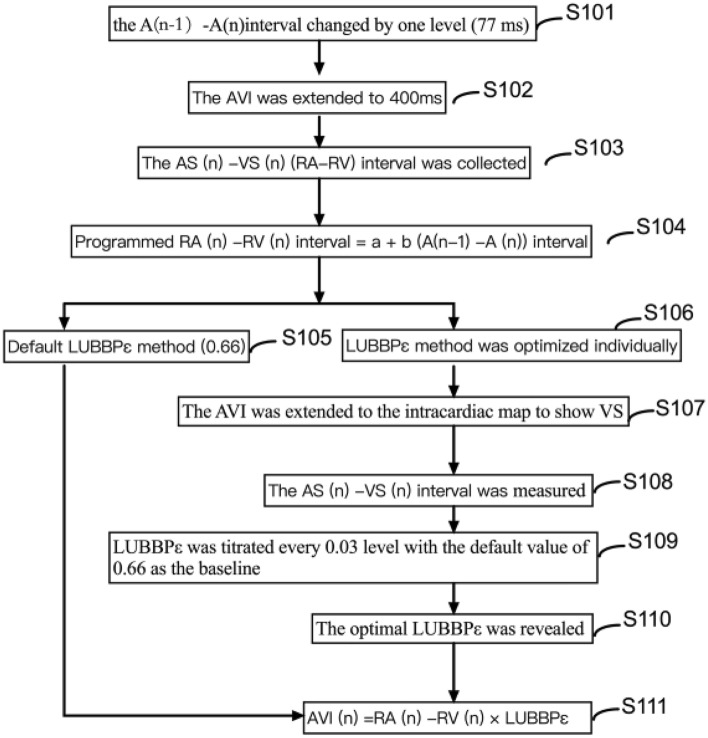
The flow chart of CRT was realized by automatically optimizing AVI based on RAS–RVS interval for single-left bundle branch pacing with three-chamber pacemaker.

① the atrial electrode was used to detect the (A) wave, with AS used as the starting point for the lower limit rate interval (LRI) (default value was 1000 ms). When the rate changed by one level (5 beats/min), the AS–AS interval changed by one level (77 ms) (S101). ② The pacemaker program automatically extended AVI to 400 ms (S102). ③ Ventricular sensing (VS) was generated by ventricular electrodes, and AS-AS and AS-VS [namely right atrial sensing and right ventricular sensing (RAS–RVS)] intervals were measured (S103), that is the RA–RV interval corresponding to the A–A interval. A total of 15 points were collected up to the LRI (the default value was 460 ms, that is, 130 ppm). ④ An algorithm was established to derive the RA–RV interval from the AS–AS interval: RA(n)–RV(n) interval = a + b [AS(n-1)-AS(n)] interval (where A is a constant term and B is a standardized partial regression coefficient, n ≥ 2). This equation can achieve an automatic calculation of the corresponding RA–RV interval when the A–A interval alters (S104). ⑤ The default LUBBPε of CRT was 0.66 (S105) with automatic AVI optimization based on the RAS–RVS interval, with the default first level of 0.03. ⑥ LUBBPε can also be set according to the empirical value after system implantation. If necessary, an individual optimization of the optimal LUBBPε (S106) can also be considered. ⑦ The AVI was extended to the intracardiac map to show VS (S107). ⑧ The AS-VS intervalwas measured (S108), acting as the RA–RV interval corresponding to the AS-AS interval ⑨ Echocardiography was optimized, and ε was titrated every 0.03 level with the default value of 0.66 as the baseline (S109). ⑩ When the ε was optimized to the maximum AVVTI and LVEF and the minimum MRA, the optimized LUBBPε was programmed into the pacemaker (S110). The regression equation of the RA–RV interval was derived from the AS-AS interval: RA(n)–RV(n) interval = a + b [AS(n − 1)  − AS(N)] interval, and the optimal AVI based on the RA–RV interval can be automatically calculated according to the optimized ε (default value 0.66), namely, AVI (n) = RA(n)  − RV(n) interval × LUBBPε (S111). The algorithm for automatic AVI optimization of a single left bundle branch of a three-chamber pacemaker based on the RA–RV interval to realize CRT can be summarized in the following schematic figure (Fig. 6).

## Discussion

In this study, the postoperative QRS duration in the single left bundle branch pacing group was shortened by an average of 44 ms in comparison to before the operation, closely reaching normal. The postoperative QRS duration in the BVP group was significantly shorter than that in the operation group. According to the comparison of the two groups, the single left bundle branch pacing group had a narrower QRS duration after the operation. This study indicated the noninferiority of single left bundle branch pacing compared to BVP in cardiac electrical synchrony and reached more consistency with physiology. During patient follow-up, ECG was adopted to program CRT to achieve the optimization of cardiac electrical synchrony. The prolonged QRS complex duration serves as a prerequisite for cardiac electrical dyssynchrony, which can be corrected by CRT. In a recent study, Jastrzebski et al. revealed a robust correlation of QRS shortening after CRT with survival of clinical heart failure endpoints, covering death^21^. Visible ECG serves as a generally practical method for observing cardiac electrical synchrony in CRT, which was evaluated as a simple and reproducible endpoint for optimizing CRT^22^.

In this study, the number of NYHA I–II in the single left bundle branch pacing group was significantly increased after surgery in comparison to before surgery, as was the number of NYHA I–II in the BVP group. However, the number of NYHA I-II in the single left bundle branch pacing group was higher than that in the standard biventricular pacing group. The postoperative 6 MWT of the single left bundle branch pacing group was significantly increased in comparison to before surgery, reaching over 450 m. The postoperative 6 MWT of the BVP group was increased, which was more significant in the single left bundle branch pacing group. This study validated the noninferior improvement in cardiac function with single-left bundle branch pacing in comparison to BVP. Studies of the acute haemodynamic effects of CRT have demonstrated that LUVP and BVP normally perform similarly and frequently better than BVP, while at least are not inferior to BVP^15–18^.

In this study, the postoperative LVEF was significantly increased in the single left bundle branch pacing group, with the postoperative LVEF being increased by approximately 5% in comparison to BVP. Cardiac stroke volume was significantly optimized, suggesting the more significant improvement of single left bundle branch pacing on cardiac function. However, no significant difference was found between the two groups, possibly resulting from the limited sample size, short follow-up time, and large individual differences in patients. In the future, the sample size can be enlarged, accompanied by an increased follow-up time. In single left bundle branch pacing group, LAD was shortened by an average of 4mm after operation. The LAD in the standard two-compartment group improved after operation. Comparison between the two groups: The single left bundle pacing group had a smaller postoperative LAD. The average postoperative LVEDD shortening in the single left bundle pacing group was 5 mm. Comparison between the two groups: The single left bundle pacing group had a shorter postoperative LVEDD. Demonstrating that single left bundle branch pacing can better reverse ventricular remodelling. The AVVTI in the single left bundle pacing group increased by 6ms after surgery, which is an improvement compared to the standard biventricular group. In conclusion, based on the cardiac ultrasound indicators, the atria and ventricles of the two groups were reduced to diverse degrees, accompanied by an increased AVVTI, indicating obvious curative effects on both groups in the treatment of heart failure. However, in the comparison of the two groups, LVEDD was only statistically significant in the single left bundle branch pacing group, probably due to the limited sample size and short follow-up time of this study. The LV electrode of the single left bundle branch pacing is located on the normal conduction system of the heart without negative influence on the myocardium. In contrast, the BVP has electrodes located in the lateral or posterior veins of the heart, leading to remodelling of the left ventricular myocardium. Synchronization index: Atrioventricular synchronicity: The atrioventricular synchronization was significantly improved in both groups, with the single left bundle branch pacing group noninferior to the BVP group. Interventricular synchronization: The interventricular synchrony of the two groups was significantly modified after the operation, with the interventricular synchrony of the single left bundle branch pacing group returning to normal. Left ventricular synchrony: Left ventricular synchrony was slightly optimized after the operation in both groups. In the synchrony index, there were significant improvements in atrioventricular, interventricular and left ventricular for both groups, indicating a significant role of CRT in the treatment of cardiac synchrony. Despite no significant difference in the synchrony index between the two groups, a significant effect was achieved in the single left bundle branch pacing group regarding the interventricular synchrony, which had returned to normal. This may be explained by (1) the left ventricular electrode being located in the left bundle branch area, constituting the normal conduction system of the heart; (2) the normal right ventricular excitation being transmitted to the good fusion of left ventricular pacing excitation. Echocardiography is frequently adopted to estimate LVEF but can also evaluate mechanical dyssynchrony of the heart, providing significant evidence for atrioventricular, interventricular and intraventricular dyssynchrony. Compared with ECG, the visual representation of the cardiac systolic process and noninvasive haemodynamic function assessment can contribute to more accurately assessing the severity of systolic dyssynchrony, which can help to better screen patients meeting the indications of CRT and apply to the optimization of CRT during follow-up^23,24^.

However, the adaptive CRT algorithm is rooted in conventional CRT, in which LV electrodes are generally implanted in the lateral or posterior veins of the left ventricle, yielding an AVI equal to 70% of the native AV interval. Because the LV electrode of LBBP is implanted below the point of LBB block and located in the conduction system of the heart, the ratio of the optimal AVI to the inherent AV interval will change, but the specific value remains unproposed. LUBBPε = 0.66 ± 0.06 was defined as the ratio of optimal AVI to intrinsic AVD (optimized AVI to RAS–RVS). The results illustrated that CRT can be achieved by optimizing AVI based on the RAS–RVS interval in a three-chamber pacemaker with single left bundle branch pacing, with the optimal AVI reaching 66% of the RAS–RVS interval. Based on the current V–V interval program of three-chamber pacemakers, one step per 10 ms, a three-chamber single left bundle branch pacing system with automatic AVI optimization was developed. Accordingly, it is proposed to develop a single left bundle branch pacing system for a three-chamber pacemaker with the capability to automatically optimize AVI based on the RA–RV interval, with the physiology of the atrioventricular node and defibrillation function retained, and perform defibrillation function in the event of ventricular tachycardia or ventricular fibrillation. Therefore, in the future, a three-chamber pacemaker can be developed and applied to a single left bundle branch pacing algorithm, which can not only realize automatic interval optimization but also ensure the safety of patients, showing obvious significance and application value in the treatment of HF.

## Conclusion

The three-chamber pacemaker for single left bundle branch pacing can achieve cardiac resynchronization therapy based on right atrial and right ventricular interval, with the optimal AVI to be 66% of RAS–RVS interval.

## Data Availability

The datasets used and/or analysed during the current study available from the corresponding author on reasonable request.
